# Development of Quantitative Real-Time PCR Assays for Rapid and Sensitive Detection of Two* Badnavirus* Species in Sugarcane

**DOI:** 10.1155/2018/8678242

**Published:** 2018-08-06

**Authors:** Sheng-Ren Sun, Kashif Ahmad, Xiao-Bin Wu, Jian-Sheng Chen, Hua-Ying Fu, Mei-Ting Huang, San-Ji Gao

**Affiliations:** ^1^National Engineering Research Center for Sugarcane, Fujian Agricultural and Forestry University, Fuzhou 350002, China; ^2^Guangzhou Sugarcane Industry Research Institute, Guangzhou 510316, Guangdong, China

## Abstract

Sugarcane-infecting badnaviruses (sugarcane bacilliform viruses, SCBVs) represent a genetically heterogeneous species complex, posing a serious threat to the yield and quality of sugarcane in all major producing regions. SCBVs are commonly transmitted across regions by the exchange of sugarcane germplasm. In this study, we develop two quick, sensitive, and reliable protocols for real-time quantitative PCR (qPCR) of* Sugarcane bacilliform MO virus* (SCBMOV) and* Sugarcane bacilliform IM virus* (SCBIMV) using two sets of TaqMan probes and primers targeting the reverse transcriptase/ribonuclease H (RT/RNase H) region. The two assays had a detection limit of 100 copies of plasmid DNA and were 100 times more sensitive than conventional PCR. High specificity of the two assays was observed with respect to SCBIMV and SCBMOV. A total of 176 sugarcane leaf tissue samples from Fujian and Yunnan provinces were collected and analyzed in parallel by conventional PCR, SCBIMV-qPCR, and SCBMOV-qPCR. The SCBIMV-qPCR and SCBMOV-qPCR assays indicated that 50% (88/176) and 47% (83/176) samples tested positive, respectively, whereas only 29% (51/176) tested positive with conventional PCR with the primer pairs SCBV-F and SCBV-R. We demonstrate for the first time that SCBIMV and SCBMOV occur in China and reveal coinfection of both* Badnavirus* species in 29% (51/176) of tested leaf samples. Our findings supply sensitive and reliable qPCR assays for the detection and quantitation of SCBV in sugarcane quarantine programs.

## 1. Introduction

Sugarcane bacilliform virus (SCBV, genus* Badnavirus*, family* Caulimoviridae*) was reported for the first time in 1985 in Cuba [1]. SCBV is distributed worldwide and threatens yield and quality of sugarcane as a result of global exchange of sugarcane germplasm [2, 3]. SCBV can infect sugarcane (*Saccharum* spp.),* Sorghum halepense*,* Brachiaria* sp.,* Panicum maximum*, and* Rottboellia exaltata* in a semipersistent manner via the insect vectors pink sugarcane mealy bug (*Saccharicoccus sacchari*) and gray sugarcane mealy bug (*Dysmicoccus boninsis*). It can also mechanically infect rice (*Oryza sativa*), banana (*Musa *sp.), and sorghum (*Sorghum vulgare* L.) using partially purified extracts or by* Agrobacterium*-mediated inoculation, but not by use of cutting implements or machinery [4–6]. SCBV is transmitted by vegetative propagation with long-distance movement across the cane-planting geographic regions [5]. Leaf freckle disease is caused by SCBV with symptoms of mottling, chlorosis, and stunted growth, with foliar symptoms varying across different host* Saccharum* and viral strains [5–7].

SCBV viral particles are nonenveloped and bacilliform 30 nm × (130-150) nm and contain a circular dsDNA genome 7.5-8.0 kb in length [4, 8]. The SCBV genome possesses typical* Badnavirus* genomic characteristics, with three open reading frames (ORFs). The precise functions of ORF1 and ORF2 are not known, while the large polyprotein encoded by ORF3 is proposed to use a viral aspartic protease to produce functional or construct proteins including a movement protein, a capsid protein, an aspartic protease, a reverse transcriptase (RT), and a ribonuclease H (RNase H) [4, 9, 10]. However, different SCBVs differ in their predicted sizes and the putative scanning model for P2 (ORF2-encoded) and P3 (ORF3-encoded) protein translation [8]. The conserved RT/RNase H domains and sequences have been frequently used for viral detection and taxonomy in* Badnavirus* [10, 11]. SCBVs exhibit high genetic variability in the viral genome [3] and serological heterogeneity [5]. A total of 18 SCBV phylogroups (A-R) have been proposed using phylogenetic grouping based on the partial RT/RNase H sequence [3, 12], the promoter region [13], or the full genome [8, 14, 15]. At present, twelve full genomic sequences of SCBV isolates have been reported from Morocco (sugarcane bacilliform MO virus-Morocco, SCBMOV-MOR) [4], Australia (sugarcane bacilliform IM virus-Queensland, SCBIMV-QLD) [9], Guadeloupe (SCBGAV-R570, SCBGAV-R51129, and SCBGDV-Batavia) [14], India (SCBV-BT, SCBV-BRU, SCBV-BO91, SCBV-Iscam, and SCBV-BB) [15], and China (SCBV-CHN1 and SCBV-CHN2) [8]. However, only* Sugarcane bacilliform MO virus* (SCBMOV),* Sugarcane bacilliform IM virus* (SCBIMV),* Sugarcane bacilliform Guadeloupe A virus* (SCBGAV), and* Sugarcane bacilliform Guadeloupe D virus* (SCBGDV) were assigned by the International Committee on Taxonomy of Viruses (ICTV) as four different species in the* Badnavirus* genus [10, 16, 17]. In addition, numerical SCBV species from different geographic origins worldwide were proposed [3, 14, 15], but this remains unclear.

SCBVs are difficult to diagnose in sugarcane in the field due to the lack of specific foliar symptoms and similar mosaic symptoms to those caused by other viruses, such as* Sugarcane mosaic virus* (SCMV),* Sorghum mosaic virus* (SrMV), and* Sugarcane streak mosaic virus* (SCSMV) [1, 18]. Various detection methods have been developed including use of electron microscopy, immunosorbent electron microscopy (IEM), enzyme linked immunosorbent assay (ELISA), polymerase chain reaction (PCR), and immunocapture PCR (IC-PCR) [3, 5]. The electron microscopy method requires expensive equipment and is slow and tedious, whereas the ELISA assay requires high quality antisera and lacks sufficient sensitivity to detect the virus in plants with a low viral titer [5, 19, 20]. PCR assays are widely used to test for SCBV because they are rapid, convenient, and more sensitive than electron microscopy or serological tests. As early as 1995, a pair of specific primers SCBV-F5/R5 targeted at the RT/RNase H region was designed for SCBV detection [21]. Subsequently, Yang et al. (2003) designed a pair of common primers BadnaFP and BadnaRP for detection of badnaviruses [22]. However, this set of primers resulted in some false positives and failed to detect certain strains [21, 22]. Recently, our previous work reported a novel set of specific primers SCBV-F and SCBV-R for more effective and extensive detection of SCBV [3]. A real-time quantitative PCR (qPCR) assay is now commonly applied to diagnose and quantify plant pathogens, including numerous disease agents in sugarcane, as this method is more efficient and reliable, but qPCR has not yet been reported for use in SCBV detection.

A few studies have investigated the occurrence and distribution of SCBVs in China using conventional PCR [3, 23, 24], whereas in this study we describe the first use of TaqMan-based qPCR assays for the quick and quantitative detection of two SCBV species (SCBIMV and SCBMOV). We use two protocols of TaqMan-based qPCR for SCBV detection and identification in 176 sugarcane leaf samples collected from fields in Fujian and Yunnan provinces, China. The two novel qPCR assays can be used in the phytosanitary management of these SCBV diseases.

## 2. Materials and Methods

### 2.1. Leaf Collection

A total of 114 sugarcane leaf samples were collected from 38 sugarcane clones (three randomly chosen leaves from different plants in each clone) in Fuzhou, Fujian province, China, in 2015. These sugarcane clones were originally imported from Chinese sugarcane research institutes in different provinces and then cultivated in Fuzhou. Another set of 62 leaf samples (one leaf sample for each cultivar in a field) was collected from three sugarcane planting regions (Baoshang, Dehong, and Lincang) in Yunnan province, China, in 2017 (Table S1). All leaf samples were rinsed with 75% ethanol and stored at −80°C until DNA extraction.

### 2.2. DNA Isolation and Purification

Total DNA of leaf tissue samples were extracted and purified using the cetyltrimethylammonium bromide (CTAB) method. All DNA samples were eluted in 50* μ*L sterile water and stored at −20°C. The quality of DNA was analyzed using a 1% agarose gel electrophoresis and concentration was measured with a Synergy™ H1 Hybrid Multimode Reader (BioTek, Winooski, VT, USA). All DNA samples were diluted into a working concentration of 100 ng/*μ*L for further SCBV detection.

### 2.3. Primers and Probes Design

Two sets of specific primers and TaqMan fluorescence probes were designed according to the RT/RNase H regions of two SCBV isolates, SCBIMV-QLD (GenBank accession number NC_003031) and SCBMOV-MOR (GenBank accession number NC_008017), using Primer Express software. The sizes of the qPCR products were 79 bp and 85 bp, for SCBIMV and SCBMOV, respectively (Table 1). The fluorescent reporter dye (FAM) and the nonfluorescent quencher dye (Eclipse) were labeled at the 5′-end and the 3′-end of the TaqMan probes, respectively. The common SCBV-specific primer pair SCBV-F and SCBV-R targeted at the conserved RT/RNase H regions was used for conventional PCR detection [3]. All primers and probes were synthesized by Takara Bioengineering Co., Ltd. (Dalian, China).

### 2.4. Plasmid Generation

The plasmid pScBV20 (10529 bp) containing the full-length genome of SCBMOV-MOR isolate was obtained from the Olszewski Lab at the University of Minnesota. The fragment of SCBIMV-QLD RT/RNase H (1229 bp) was synthesized by Beijing Genomics Institute (Shenzhen, China) and cloned into the pMD18-T vector to obtain the 3821 bp pMD18T-IM plasmid. Similarly, other plasmids for nine SCBV genotypes, SCBGAV-R570 (FJ824813), SCBGDV-Batavia (FJ824817), SCBV-BO91 (JN377533), SCBV-Iscam (JN377534), SCBV-BB (JN377535), SCBV-BT (JN377536), SCBV-BRU (JN377537), SCBV-CHN1 (KM214357), and SCBV-CHN2 (KM214358), were synthesized and then constructed into pMD18-T vectors.

### 2.5. Conventional PCR and qPCR Assays

Conventional PCR was carried out in a 25 *μ*L mixture containing 1 *μ*L DNA, 2.5 *μ*L of 10×*EX* Taq Buffer, 0.2 mmoL/L of dNTPs, 0.4 *μ*moL/L of each of the forward and reverse primers, and 1.25 U of* EX* Taq, with sterile water added to create a final volume of 25 *μ*L. PCR amplification with SCBV-F/SCBV-R primers was performed by following a thermal cycling program of 94°C for 5 min, 35 cycles of 94°C for 30 s, 58°C for 30 s, and 72°C for 45 s, and a final extension at 72°C for 10 min. Conventional PCR amplification with SCBIMV-qPCR or SCBMOV-qPCR primers was performed following a thermal cycling program of 94°C for 2 min, 35 cycles of 94°C for 30 s, 54°C (IM-QF2/IM-QR2) or 60°C (MOR-QF2/MOR-QR2) for 30 s, and 72°C for 30 s, and a final extension at 72°C for 5 min. The FastStart Universal Probe Master Kit (Roche Applied Science, Mannheim, Germany) was used for the fluorescence qPCR assay. qPCR was carried out in a 25 *μ*L mixture containing 1 *μ*L DNA (100 ng/*μ*L), 12.5 *μ*L TaqMan Fast Universal PCR Master Mix, 2.25 *μ*L (10 pmol) of each of forward and reverse primers, and 1 *μ*L (10 pmol) TaqMan probe, with sterile water added to create a final volume of 25 *μ*L. The qPCR optimum cycling conditions consisted of 50°C for 2 min, 95°C for 10 min, 40 cycles of 95°C for 15 s, and 60°C for 1 min.

### 2.6. Standard Curve Construction for qPCR

The recombinant plasmid DNA with 10-fold serial dilutions (10^9^-100 copies/*μ*L) was used for establishing the standard curve for qPCR with an ABI 7500 Real-Time PCR System (Applied Biosystems, Foster City, CA, USA). Three biological replicates were used for each serial dilution of plasmid DNA. The amplification efficiency was calculated as* E* = (10^-1/slope^-1) × 100%.

### 2.7. Specificity and Sensitivity Evaluations for qPCR

To test the specificity of real-time qPCR, the plasmid DNA of pMD18T-IM and pScBV20 was used as positive controls for SCBIMV-qPCR and SCBMOV-qPCR, respectively. The plasmid DNA of nine other SCBV isolates (SCBGAV-R570 and SCBGDV-Batavia, SCBV-BO91, SCBV-BB, SCBV-BT, SCBV-BRU, SCBV-Iscam, SCBV-CHN1, and SCBV-CHN2) was also tested. The DNA concentration of 10^5^ copies/*μ*L was used as qPCR template for each plasmid. To assess the sensitivity of real-time qPCR and conventional PCR assays in gel electrophoresis, the recombinant plasmids pMD18T-IM and pScBV20 (10^8^-10 copies/*μ*L) were detected by conventional PCR in parallel with two sets of qPCR primers (IM-QF2/IM-QR2 and MOR-QF2/MOR-QR2) and one set of PCR primers SCBV-F/SCBV-R. The PCR products of the IM-QF2/IM-QR2 and MOR-QF2/MOR-QR2 primers were detected using 4% low melting agarose gel electrophoresis, while the PCR products of SCBV-F/SCBV-R primers were detected with 1.5% low melting agarose gel electrophoresis. The total DNA (100 ng/*μ*L) of sugarcane leaf without SCBV infection was used as a negative control and sterile water was used as a blank control in all the above-mentioned experiments.

### 2.8. SCBV Detection in Field Leaf Samples

Sugarcane leaf samples collected from the fields were detected in parallel by conventional PCR with SCBV-F/SCBV-R primers and by qPCR assays of SCBIMV and SCBMOV. All PCR products were analyzed with 1.5% agarose gel electrophoresis and then cloned into the pMD19-T vector for sequencing.

## 3. Results

### 3.1. Specificity Analysis* In Silico* of Primers and Probes

**Figure 1:**
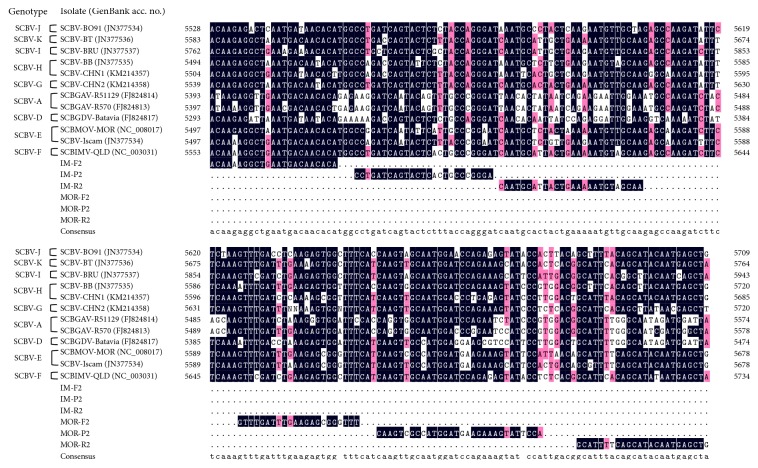
Multiple sequences alignment of nucleotide sequences between two sets of primers and probes of real-time quantitative PCR (qPCR) assays of* Sugarcane bacilliform MO virus* (SCBMOV) and* Sugarcane bacilliform IM virus* (SCBIMV) with the corresponding regions (RT/RNase H) of the 12 published sugarcane bacilliform virus (SCBV) genotypes/phylogroups.

The two sets of primers and probes of real-time qPCR for the two SCBV species (SCBIMV and SCBMOV) were evaluated* in silico* by multiple sequence alignment. The available RT/RNase H region sequences, representing 12 different SCBV genotypes/phylogroups, including SCBIMV and SCBMOV, were compared (Figure 1). Various variable nucleotides were found in the two sets of primers and probes of SCBIMV-QLD and SCBMOV-MOR by comparison of RT/RNase H sequences with one another and with the other ten SCBV genotypes. The IM-P2 probe of SCBIMV had five highly variable nucleotides and the primers IM-QF2/IM-QR2 ranged from two to seven highly variable nucleotides. The MOR-P2 probe of SCBMOV had seven highly variable nucleotides and the primers MOR-QF2/MOR-QR2 had from three to four highly variable nucleotides. The primers and probes sequences of SCBIMV-qPCR and SCBMOV-qPCR were specific with respect to individual SCBV isolate, SCBIMV-QLD or SCBMOV-MOR.

### 3.2. Amplification Efficiency Analysis of Real-Time qPCR Assay

**Figure 2:**
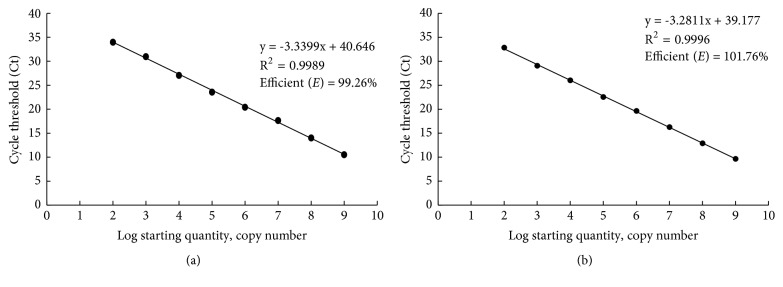
Standard curves for the real-time quantitative PCR (qPCR) assays of* Sugarcane bacilliform MO virus* (SCBMOV) and* Sugarcane bacilliform IM virus* (SCBIMV). (a) Standard curve of SCBIMV-qPCR using the templates of pMD18T-IM (10^9^-10^2^ copies/*μ*L). (b) Standard curve of SCBMOV-qPCR using the templates of pScBV20 (10^9^-10^2^ copies/*μ*L).

Two standard curves of real-time qPCR of SCBIMV and SCBMOV were established using the plasmid DNA of pMD18T-IM and pScBV20, respectively (Figure 2). The standard curve for pMD18T-IM had a slope of -3.3399, efficiency (*E*) = 99.26%, and R^2^ = 0.9989 (Figure 2(a)). The standard curve for pScBV20 had a slope of -3.2811, efficiency (*E*) = 101.76%, and R^2^ = 0.9996 (Figure 2(b)). According to the cut-off values of Ct = 35, the minimum detection limit of the real-time qPCR assay was 100 copies for plasmid DNA of both pMD18T-IM and pScBV20.

### 3.3. Sensitivity Evaluation of Real-Time qPCR Assay

**Figure 3:**
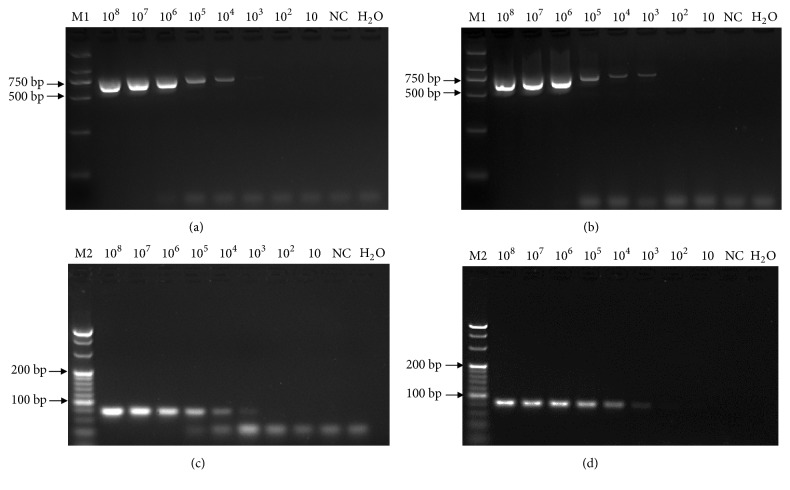
Sensitivity tests for two sets of real-time quantitative PCR (qPCR) primers of MOR-F2/MOR-R2 and IM-F2/IM-R2 and a set of conventional PCR primers SCBV-F/SCBV-R using gel electrophoresis. (a) Serial dilutions (10^8^–10 copies/*μ*L) of pMD18T-IM plasmid DNA with SCBV-F/SCBV-R primers. (b) Serial dilutions (10^8^–10 copies/*μ*L) of pScBV20 plasmid DNA with SCBV-F/SCBV-R primers. (c) Serial dilutions (10^8^–10 copies/*μ*L) of pMD18T-IM plasmid DNA with IM-F2/IM-R2 primers. (d) Serial dilutions (10^8^–10 copies/*μ*L) of pScBV20 plasmid DNA with MOR-F2/MOR-R2 primers. M1, DNA Marker DL2,000; M2, 20 bp DNA Ladder Marker; NC, total DNA (100 ng/*μ*L) of SCBV-negative sugarcane leaf; H_2_O, blank control.

To compare the sensitivity between TaqMan-based qPCR and conventional PCR assays, two sets of templates of eight tenfold serial dilutions (10^8^-10 copies/*μ*L) of pMD18T-IM and pSCBV20 plasmids were used in conventional PCR with the three pairs of primers SCBV-F/SCBV-R, IM-QF2/IM-QR2, and MOR-QF2/MOR-QR2. The expected product sizes of 726 bp, 79 bp, and 85 bp were observed in gel electrophoresis for SCBV-F/SCBV-R, IM-QF2/IM-QR2, and MOR-QF2/IM-QR2, respectively (Figure 3). Conventional PCR results revealed that the minimum detection limits were 10^4^ copies/*μ*L (Figure 3(a)) and 10^3^ copies/*μ*L (Figure 3(b)) for pMD18T-IM and pScBV20 plasmid DNA, respectively, using the primer pair SCBV-F/SCBV-R. Similarly, the minimum detection limits were 10^4^ copies/*μ*L (Figure 3(c)) for pMD18T-IM with the primers IM-QF2/QR2 and 10^4^ copies/*μ*L (Figure 3(d)) for pScBV20 with primers MOR-QF2/MOR-QR2. A minimum detection limit of 100 copies/*μ*L of pMD18T-IM and pSCBV20 plasmid DNA was obtained by qPCR methods according to the criterion of Ct≤35 as the effective detection threshold (Figure 2).

### 3.4. Specificity Evaluation of Real-Time qPCR Assay

The qPCR primers and probes for SCBIMV and SCBMOV species were assessed with one another and with other SCBV genotypes using a plasmid DNA template (10^5^ copies/*μ*L). The primers and probe of SCBIMV-qPCR were highly specific for the SCBIMV-QLD isolate and the Ct-value was 23.0, while being undetermined in other SCBV isolates including SCBMOV-MOR (Table 2). The primers and probe for SCBMOV-qPCR were also highly specific to the SCBMOV-MOR isolate, with a Ct-value of 22.1. However, two Ct values closer to 35 (34.8 and 33.6) were determined in the two Indian isolates SCBV-Iscam and SCBV-BT, and a Ct-value of 36.8 occurred in SCBV-CHN2 (Table 2). No fluorescence signals were detected in the remaining SCBV genotypes.

### 3.5. Field Sample Detection for SCBV

Of the 176 leaf samples, 50% and 47% were indicated positive by the SCBIMV-qPCR and SCBMOV-qPCR assays, respectively, while 29% of samples were positive when tested with conventional PCR (Table 3). 29% (51/176) of samples were coinfected by SCBIMV and SCBMOV. Among the Fujian leaf samples, 81/114 (71%) leaf samples from 38 sugarcane clones were positive when tested with SCBIMV-qPCR, and viral titer varied from 5.7×10 to 1.7×10^4^ copies/*μ*L, while 65/114 (57%) leaf samples were positive with SCBMOV-qPCR and viral titer varied from 3.8×10 to 5.0×10^3^ copies/*μ*L. Among Yunnan leaf samples, 7/62 (17%) leaf samples tested positive with SCBIMV-QPCR and viral titer varied from 64 to 110 copies/*μ*L, whereas 17/62 (27%) leaf samples tested positive with SCBMOV-QPCR and viral titer varied from 2.7×10 to 3.0 × 10^2^ copies/*μ*L. Only 46/176 (40%) and 5/176 (3%) leaf samples were positive using conventional PCR in Fujian and Yunnan provinces, respectively.

To elucidate the probability of nonspecific amplification, all products of qPCR and conventional PCR were cloned and sequenced. All the sequences were verified as SCBV with the BLASTN tool (http://blast.ncbi.nlm.nih.gov/).

## 4. Discussion

Sugarcane is an important crop, accounting for 70% of the raw sugar produced worldwide and 90% of sugar production in China. Sugarcane germplasms are frequently exchanged between and within countries to broaden the genetic base for modern commercial hybrid breeding. Thus, viruses often spread across geopolitical boundaries by taking advantage of the worldwide sugarcane germplasm exchange. Developing a rapid, efficient, and sensitive molecular detection assay plays an important role in the phytosanitary management of virus diseases. Various qPCR assays have been developed and applied in the detection of numerous plant viruses and pathogenic bacteria or fungi causing major economic losses in the global agricultural industry. Commercially accessible qPCR methods include SYBR Green, TaqMan, Scorpions, and molecular beacons, with TaqMan being the most utilized in real-time fluorescent detection systems, because TaqMan-based qPCR can discriminate between sequences that differ by only a single nucleotide [25]. Real-time qPCR assays based on TaqMan probes have been developed for quick and quantitative detection of various disease agents in sugarcane, but there had not been any report of SCBV detection by qPCR.

The RT/RNase H region in the viral genome of* Badnavirus *is commonly used for PCR detection, phylogenetic analysis, and viral taxonomy [11, 22]. Partial RT/RNase H sequences have been used as taxonomic markers for addressing the sequence diversity among SCBV isolates [3, 12–14]. However, SCBV complexes pose high genetic variation and were clustered into at least 18 genotypes/phylogroups [3]. It is difficult to design a universal primer with TaqMan probe for diagnosis of SCBVs. At present, four ICTV-recognized SCBV species are known to be associated with sugarcane leaf fleck disease around the world, whereas their geographic distributions tended to be related to origins in some extent. For instance, SCBGAV (SCBV-A group) was distributed in Guadeloupe and Brazil, but SCBGDV (SCBV-D group) was only distributed in Guadeloupe [14, 26]; SCBMOV (SCBV-E group) was presented in Morocco [4] and India [15], but SCBIMV (SCBV-F group) occurred in Australia [9] and Brazil [26]. In this study, we designed two sets of primers and TaqMan probes for developing real-time qPCR assays for the two* Badnavirus* species SCBIMV and SCBMOV. To our knowledge, this is the first time that real-time TaqMan qPCR assays for detection and quantification of SCBIMV and SCBMOV have been developed.

Significant and high sensitivity and specificity are the most important features of a qPCR assay. Our results demonstrated that the sensitivity of SCBIMV-qPCR or SCBMOV-qPCR was 1000-fold greater than that of conventional PCR. In addition, this elevated sensitivity and efficiency were also demonstrated in the field samples of sugarcane leaves. Similar results were found in other studies of viral pathogen detection in sugarcane [27]. In the current study, we evaluated the specificity of SCBIMV-qPCR or SCBMOV-qPCR assays by testing in parallel the plasmid DNA representing different SCBV genotypes/phylogroups. The results suggested the assays were highly specific for SCBIMV-qPCR or SCBMOV-qPCR. The specificity of qPCR probes and primers was also verified* in silico* through multiple sequence alignment. Although the Ct-values of SCBV-Iscam and SCBV-BT plasmid DNA were very close to 35 (the effective detection threshold) using the SCBMOV-qPCR assay, this may have resulted from SCBV-Iscam and SCBMOV-MOR sharing high similarity and clustering into the same genotype (SCBV-E) [15]. Further work in our lab will develop the specific primers and probes for other SCBV species/genotypes.

Samples from Fuzhou, Fujian province, tested positive for SCBV more frequently than those from Yunnan provinces using either qPCR or conventional PCR. One reason for this observed pattern could be that the SCBVs were introduced into Fuzhou from other provinces by SCBV-infected vegetative cuttings imported for national sugarcane regional tests (Table S1). These samples could have also been infected by existing SCBVs in Fuzhou during the cultivating period. Numerous SCBV genotypes have been reported in the Chinese sugarcane planting regions with different levels of incidence [3], whereas in this study SCBMOV and SCBIMV were detected for the first time in China using qPCR assays. Some experiments indicated that heat treatment and tissue culture were ineffective in eliminating SCBV from* Saccharum* spp. clones, which increases challenges for the prevention and control of this disease [5, 28]. Hence, there is an urgent need to prevent SCBV transmission across sugarcane producing provinces or counties. Healthy germplasms and cuttings can be identified using a reliable, sensitive, and efficient qPCR or multiplex qPCR assay.

## 5. Conclusion

Here, two TaqMan-based qPCR methods were developed for viral detection of two SCBV species SCBIMV and SCBMOV for the first time. Our findings revealed that the two assays, SCBIMV-qPCR and SCBMOV-qPCR, are more robust, sensitive, and efficient for the detection and quantification of SCBV and can be used for screening of sugarcane germplasm. Our findings provide support for detection, control, and monitoring of SCBV via healthy seed cane certification and will also be helpful for accurate measurement of the occurrence of SCBIMV and SCBMOV in commercial sugarcane fields.

## Figures and Tables

**Table 1:** A list of primers and TaqMan probes used in this study for detecting sugarcane bacilliform viruses (SCBVs) infecting sugarcane.

Primer/Probe	Virus detection	Sequence (5′⟶3′)^a^	Size of fragment (bp)	Reference
SCBV-F	SCBVs	GTTCATCGCHGTNTAYATTGATGAC	726	Wu et al., 2016 [3]
SCBV-R		GAAGGYTTRTGTTCTVCACTCTTGTTG		
IM-QF2	SCBIMV	ACAAAAGGCTGAATGACAACACA	79	this study
IM-QR2		TTGCTACATTTTTCAGTAATGCATTG		
IM-QP2		FAM-CCTGATCAGTACTCACTGCCCGGGA-Eclipse		
MOR-QF2	SCBMOV	CAGCTCATTGTATGCTGAAAATGC	85	this study
MOR-QR2		GTTTGATTTGAAGAGCGGGTTT		
MOR-QP2		FAM-TGGAATACTTTCTTCATCCATGGCGACTTG-Eclipse		

^a^Y = C/T, H = A/C/T, R = A/G, V = A/G/C, and N = A/G/C/T in primer sequences. TaqMan probes (IM-QP2 and MOR-QP2) were labeled with fluorescent reporter dye (FAM) at 5′-end and nonfluorescent quencher dye (Eclipse) at the 3′-end.

**Table 2:** Specificity tests of real-time quantitative PCR (qPCR) assays of *Sugarcane bacilliform MO virus* (SCBMOV) and *Sugarcane bacilliform IM virus* (SCBIMV) with the plasmids of other published sugarcane bacilliform virus (SCBV) genotypes/phylogroups.

Isolate	Genotype	GenBank acc. no.	SCBIMV-qPCR^a^	SCBMOV-qPCR^a^
SCBGAV-R570	SCBV-A	FJ824813	- (nd)	- (nd)
SCBGDV-Batavia	SCBV-D	FJ824817	- (nd)	- (nd)
SCBV-Iscam	SCBV-E	JN377534	- (nd)	+ (34.8)
SCBMOV-MOR	SCBV-E	NC_008017	- (nd)	+ (22.1)
SCBIMV-QLD	SCBV-F	NC_003031	+ (23.0)	- (nd)
SCBV-CHN2	SCBV-G	KM214358	- (nd)	- (nd)
SCBV-CHN1	SCBV-H	KM214357	- (nd)	- (36.8)
SCBV-BB	SCBV-H	JN377535	- (nd)	- (nd)
SCBV-BRU	SCBV-I	JN377537	- (nd)	- (nd)
SCBV-BO91	SCBV-J	JN377533	- (nd)	- (nd)
SCBV-BT	SCBV-K	JN377536	- (nd)	+ (33.6)

^a^Positive result (+) if Ct ≤ 35, and negative result (−) if Ct > 35; Ct value or data not determined (nd) shown in brackets.

**Table 3:** Detection of sugarcane bacilliform viruses (SCBVs) in sugarcane leaf samples from Fujian and Yunnan provinces using real-time quantitative PCR (qPCR) and conventional PCR.

Location	No. of sugarcane varieties tested	No. of leaf samples tested	qPCR					Conventional PCR
			SCBIMV	Copies/*µ*L	SCBMOV	Copies/*µ*L	Coinfection	
Fuzhou, Fujian	38	114	81 (71%)	5.7×10^1^-1.7×10^4^	66 (58%)	3.8×10^1^-1.5×10^3^	47 (41%)	46 (40%)
Boshang, Yunnan	7	8	0	0	3 (38%)	3.4×10^1^-7.1×10^1^	0	0
Dehong, Yunnan	13	13	0	0	2 (15%)	2.7×10^1^-3.8×10^1^	0	1 (8%)
Lingcang, Yunnan	35	41	7 (17%)	6.4×10^1^-1.1×10^2^	12 (29%)	1.8×10^1^-3.0×10^2^	4 (10%)	4 (10%)
Total	93	176	88 (50%)	0	83 (47%)	0	51 (29%)	51 (29%)

## Data Availability

The data used to support the findings of this study are included within the article and its additional files.

## Supplementary Materials

Supplementary Materials Table S1: list of sugarcane leaf samples collected from Fujian and Yunnan provinces, China.
