# Supplementary material for: Development of Quantitative Real-Time PCR Assays for Rapid and Sensitive Detection of Two Badnavirus Species in Sugarcane
Source: Biomed Res Int. 2018 Aug 6;2018:8678242. doi: 10.1155/2018/8678242 (PMC6106854; doi:10.1155/2018/8678242)
Supplement: Supplementary Materials — Table S1: list of sugarcane leaf samples collected from Fujian and Yunnan provinces, China. [file 8678242.f1.pdf]

TABLE S1 List of sugarcane leaf samples collected from Fujian and Yunnan provinces, China

No.	Clone [a]	Sampling date	Sampling location	Geographic origin
1	FN07-2020(1)	2015/06	Fuzhou, Fujian	Fuzhou, Fujian
2	FN07-2020(2)	2015/06	Fuzhou, Fujian	Fuzhou, Fujian
3	FN07-2020(3)	2015/06	Fuzhou, Fujian	Fuzhou, Fujian
4	FN40(1)	2015/06	Fuzhou, Fujian	Fuzhou, Fujian
5	FN40(2)	2015/06	Fuzhou, Fujian	Fuzhou, Fujian
6	FN40(3)	2015/06	Fuzhou, Fujian	Fuzhou, Fujian
7	GZ07-538(1)	2015/06	Fuzhou, Fujian	Ganzhou, Jiangxi
8	GZ07-538(2)	2015/06	Fuzhou, Fujian	Ganzhou, Jiangxi
9	GZ07-538(3)	2015/06	Fuzhou, Fujian	Ganzhou, Jiangxi
10	LC07-500(1)	2015/06	Fuzhou, Fujian	Liuzhou, Guangxi
11	LC07-500(2)	2015/06	Fuzhou, Fujian	Liuzhou, Guangxi
12	LC07-50(3)	2015/06	Fuzhou, Fujian	Liuzhou, Guangxi
13	MT02-205(1)	2015/06	Fuzhou, Fujian	Zhangzhou, Fujian
14	MT02-205(2)	2015/06	Fuzhou, Fujian	Zhangzhou, Fujian
15	MT02-205(3)	2015/06	Fuzhou, Fujian	Zhangzhou, Fujian
16	ROC16(1)	2015/06	Fuzhou, Fujian	Fuzhou, Fujian
17	ROC16(2)	2015/06	Fuzhou, Fujian	Fuzhou, Fujian
18	ROC16(3)	2015/06	Fuzhou, Fujian	Fuzhou, Fujian
19	YG43(1)	2015/06	Fuzhou, Fujian	Guangzhou, Guangdong
20	YG43(2)	2015/06	Fuzhou, Fujian	Guangzhou, Guangdong
21	YG43(3)	2015/06	Fuzhou, Fujian	Guangzhou, Guangdong
22	YG46(1)	2015/06	Fuzhou, Fujian	Guangzhou, Guangdong
23	YG46(2)	2015/06	Fuzhou, Fujian	Guangzhou, Guangdong
24	YG46(3)	2015/06	Fuzhou, Fujian	Guangzhou, Guangdong
25	YR07-1433(1)	2015/06	Fuzhou, Fujian	Ruili, Yunnan
26	YR07-1433(2)	2015/06	Fuzhou, Fujian	Ruili, Yunnan
27	YR07-1433(3)	2015/06	Fuzhou, Fujian	Ruili, Yunnan
28	YZ07-2384(1)	2015/06	Fuzhou, Fujian	Kaiyuan, Yunnan
29	YZ07-2384(2)	2015/06	Fuzhou, Fujian	Kaiyuan, Yunnan
30	YZ07-2384(3)	2015/06	Fuzhou, Fujian	Kaiyuan, Yunnan
31	DZ07-36(1)	2015/06	Fuzhou, Fujian	Dehong, Yunnan
32	DZ07-36(2)	2015/06	Fuzhou, Fujian	Dehong, Yunnan
33	DZ07-36(3)	2015/06	Fuzhou, Fujian	Dehong, Yunnan
34	FN09-2201(1)	2015/06	Fuzhou, Fujian	Fuzhou, Fujian
35	FN09-2201(2)	2015/06	Fuzhou, Fujian	Fuzhou, Fujian
36	FN09-2201(3)	2015/06	Fuzhou, Fujian	Fuzhou, Fujian
37	FN09-7111(1)	2015/06	Fuzhou, Fujian	Fuzhou, Fujian
38	FN09-7111(2)	2015/06	Fuzhou, Fujian	Fuzhou, Fujian
39	FN09-7111(3)	2015/06	Fuzhou, Fujian	Fuzhou, Fujian
40	GT06-2081(1)	2015/06	Fuzhou, Fujian	Nanning, Guangxi
41	GT06-2081(2)	2015/06	Fuzhou, Fujian	Nanning, Guangxi
42	GT06-2081(3)	2015/06	Fuzhou, Fujian	Nanning, Guangxi
43	GT07-25(1)	2015/06	Fuzhou, Fujian	Nanning, Guangxi
44	GT07-25(2)	2015/06	Fuzhou, Fujian	Nanning, Guangxi
45	GT07-25(3)	2015/06	Fuzhou, Fujian	Nanning, Guangxi
46	GT08-1180(1)	2015/06	Fuzhou, Fujian	Nanning, Guangxi
47	GT08-1180(2)	2015/06	Fuzhou, Fujian	Nanning, Guangxi

48	GT08-1180(3)	2015/06	Fuzhou, Fujian	Nanning, Guangxi
49	Haizhe22(1)	2015/06	Fuzhou, Fujian	Yacheng, Hainan
50	Haizhe22(2)	2015/06	Fuzhou, Fujian	Yacheng, Hainan
51	Haizhe22(3)	2015/06	Fuzhou, Fujian	Yacheng, Hainan
52	LC07-150(1)	2015/06	Fuzhou, Fujian	Liuzhou, Guangxi
53	LC07-150(2)	2015/06	Fuzhou, Fujian	Liuzhou, Guangxi
54	LC07-150(3)	2015/06	Fuzhou, Fujian	Liuzhou, Guangxi
55	ROC22(1)	2015/06	Fuzhou, Fujian	Taiwan Island
56	ROC22(2)	2015/06	Fuzhou, Fujian	Taiwan Island
57	ROC22(3)	2015/06	Fuzhou, Fujian	Taiwan Island
58	YG47(1)	2015/06	Fuzhou, Fujian	Guangzhou, Guangdong
59	YG47(2)	2015/06	Fuzhou, Fujian	Guangzhou, Guangdong
60	YG47(3)	2015/06	Fuzhou, Fujian	Guangzhou, Guangdong
61	YZ08-1145(1)	2015/06	Fuzhou, Fujian	Kaiyuan, Yunnan
62	YZ08-1145(2)	2015/06	Fuzhou, Fujian	Kaiyuan, Yunnan
63	YZ08-1145(3)	2015/06	Fuzhou, Fujian	Kaiyuan, Yunnan
64	FN10-6716(1)	2015/06	Fuzhou, Fujian	Fuzhou, Fujian
65	FN10-6716(2)	2015/06	Fuzhou, Fujian	Fuzhou, Fujian
66	FN10-6716(3)	2015/06	Fuzhou, Fujian	Fuzhou, Fujian
67	DZ09-78(1)	2015/06	Fuzhou, Fujian	Dehong, Yunnan
68	DZ09-78(2)	2015/06	Fuzhou, Fujian	Dehong, Yunnan
69	DZ09-78(3)	2015/06	Fuzhou, Fujian	Dehong, Yunnan
70	FN09-12206(1)	2015/06	Fuzhou, Fujian	Fuzhou, Fujian
71	FN09-12206(2)	2015/06	Fuzhou, Fujian	Fuzhou, Fujian
72	FN09-12206(3)	2015/06	Fuzhou, Fujian	Fuzhou, Fujian
73	FN09-4095(1)	2015/06	Fuzhou, Fujian	Fuzhou, Fujian
74	FN09-4095(2)	2015/06	Fuzhou, Fujian	Fuzhou, Fujian
75	FN09-4095(3)	2015/06	Fuzhou, Fujian	Fuzhou, Fujian
76	FN10-2105(1)	2015/06	Fuzhou, Fujian	Fuzhou, Fujian
77	FN10-2105(2)	2015/06	Fuzhou, Fujian	Fuzhou, Fujian
78	FN10-2105(3)	2015/06	Fuzhou, Fujian	Fuzhou, Fujian
79	GT06-1492(1)	2015/06	Fuzhou, Fujian	Nanning, Guangxi
80	GT06-1492(2)	2015/06	Fuzhou, Fujian	Nanning, Guangxi
81	GT06-1492(3)	2015/06	Fuzhou, Fujian	Nanning, Guangxi
82	GT08-1533(1)	2015/06	Fuzhou, Fujian	Nanning, Guangxi
83	GT08-1533(2)	2015/06	Fuzhou, Fujian	Nanning, Guangxi
84	GT08-1533(3)	2015/06	Fuzhou, Fujian	Nanning, Guangxi
85	LC07-506(1)	2015/06	Fuzhou, Fujian	Liuzhou, Guangxi
86	LC07-506(2)	2015/06	Fuzhou, Fujian	Liuzhou, Guangxi
87	LC07-506(3)	2015/06	Fuzhou, Fujian	Liuzhou, Guangxi
88	MT07-2005(1)	2015/06	Fuzhou, Fujian	Zhangzhou, Fujian
89	MT07-2005(2)	2015/06	Fuzhou, Fujian	Zhangzhou, Fujian
90	MT07-2005(3)	2015/06	Fuzhou, Fujian	Zhangzhou, Fujian
91	MT09-104(1)	2015/06	Fuzhou, Fujian	Zhangzhou, Fujian
92	MT09-104(2)	2015/06	Fuzhou, Fujian	Zhangzhou, Fujian
93	MT09-104(3)	2015/06	Fuzhou, Fujian	Zhangzhou, Fujian
94	YG48(1)	2015/06	Fuzhou, Fujian	Guangzhou, Guangdong
95	YG48(2)	2015/06	Fuzhou, Fujian	Guangzhou, Guangdong
96	YG48(3)	2015/06	Fuzhou, Fujian	Guangzhou, Guangdong
97	YG50(1)	2015/06	Fuzhou, Fujian	Guangzhou, Guangdong

98	YG50(2)	2015/06	Fuzhou, Fujian	Guangzhou, Guangdong
99	YG50(3)	2015/06	Fuzhou, Fujian	Guangzhou, Guangdong
100	YR09-315(1)	2015/06	Fuzhou, Fujian	Ruili, Yunnan
101	YR09-315(2)	2015/06	Fuzhou, Fujian	Ruili, Yunnan
102	YR09-315(3)	2015/06	Fuzhou, Fujian	Ruili, Yunnan
103	YR10-187(1)	2015/06	Fuzhou, Fujian	Ruili, Yunnan
104	YR10-187(2)	2015/06	Fuzhou, Fujian	Ruili, Yunnan
105	YR10-187(3)	2015/06	Fuzhou, Fujian	Ruili, Yunnan
106	YR10-701(1)	2015/06	Fuzhou, Fujian	Ruili, Yunnan
107	YR10-701(2)	2015/06	Fuzhou, Fujian	Ruili, Yunnan
108	YR10-701(3)	2015/06	Fuzhou, Fujian	Ruili, Yunnan
109	YZ09-1208(1)	2015/06	Fuzhou, Fujian	Kaiyuan, Yunnan
110	YZ09-1208(2)	2015/06	Fuzhou, Fujian	Kaiyuan, Yunnan
111	YZ09-1208(3)	2015/06	Fuzhou, Fujian	Kaiyuan, Yunnan
112	YZ09-1601(1)	2015/06	Fuzhou, Fujian	Kaiyuan, Yunnan
113	YZ09-1601(2)	2015/06	Fuzhou, Fujian	Kaiyuan, Yunnan
114	YZ09-1601(3)	2015/06	Fuzhou, Fujian	Kaiyuan, Yunnan
115	FN09-12206	2017/07	Baoshan, Yunnan	Fuzhou, Fujian
116	FN11-2097	2017/07	Baoshan, Yunnan	Fuzhou, Fujian
117	YZ10-1666	2017/07	Baoshan, Yunnan	Kaiyuan, Yunnan
118	YR11-450	2017/07	Baoshan, Yunnan	Ruili, Yunnan
119	YZ11-3898	2017/07	Baoshan, Yunnan	Kaiyuan, Yunnan
120	ROC22	2017/07	Baoshan, Yunnan	Taiwan Island
121	ROC22	2017/07	Baoshan, Yunnan	Taiwan Island
122	YT86-368	2017/07	Baoshan, Yunnan	Guangzhou, Guangdong
123	FN09-12206	2017/07	Dehong, Yunnan	Fuzhou, Fujian
124	FN09-6201	2017/07	Dehong, Yunnan	Fuzhou, Fujian
125	FN09-2201	2017/07	Dehong, Yunnan	Fuzhou, Fujian
126	GT06-1492	2017/07	Dehong, Yunnan	Nanning, Guangxi
127	GT08-120	2017/07	Dehong, Yunnan	Nanning, Guangxi
128	YZ08-1095	2017/07	Dehong, Yunnan	Kaiyuan, Yunnan
129	YZ11-120	2017/07	Dehong, Yunnan	Kaiyuan, Yunnan
130	MT11-610	2017/07	Dehong, Yunnan	Zhangzhou, Fujian
131	Zhongtang1201	2017/07	Dehong, Yunnan	Danzhou, Hainan
132	Zhongzhe10	2017/07	Dehong, Yunnan	Nanning, Guangxi
133	GT08-8	2017/07	Dehong, Yunnan	Nanning, Guangxi
134	YZ10-1666	2017/07	Dehong, Yunnan	Kaiyuan, Yunnan
135	YT93-159	2017/07	Dehong, Yunnan	Guangzhou, Guangdong
136	YG47	2017/07	Lincang, Yunnan	Guangzhou, Guangdong
137	GT06-2081	2017/07	Lincang, Yunnan	Nanning, Guangxi
138	GT08-1180	2017/07	Lincang, Yunnan	Nanning, Guangxi
139	YZ08-1095	2017/07	Lincang, Yunnan	Kaiyuan, Yunnan
140	ROC22	2017/07	Lincang, Yunnan	Taiwan Island
141	FN09-2201	2017/07	Lincang, Yunnan	Fuzhou, Fujian
142	LC07-150	2017/07	Lincang, Yunnan	Liuzhou, Guangxi
143	DZ07-36	2017/07	Lincang, Yunnan	Dehong, Yunnan
144	MT06-1405	2017/07	Lincang, Yunnan	Zhangzhou, Fujian
145	GT06-1492	2017/07	Lincang, Yunnan	Nanning, Guangxi
146	MT07-2005	2017/07	Lincang, Yunnan	Zhangzhou, Fujian
147	Zhongtang1201	2017/07	Lincang, Yunnan	Danzhou, Hainan

148	Zhongzhe1	2017/07	Lincang, Yunnan	Nanning, Guangxi
149	YZ10-1666	2017/07	Lincang, Yunnan	Kaiyuan, Yunnan
150	GT08-8	2017/07	Lincang, Yunnan	Nanning, Guangxi
151	GT10-701	2017/07	Lincang, Yunnan	Nanning, Guangxi
152	YZ11-3898	2017/07	Lincang, Yunnan	Kaiyuan, Yunnan
153	GT08-120	2017/07	Lincang, Yunnan	Nanning, Guangxi
154	FN09-7111	2017/07	Lincang, Yunnan	Fuzhou, Fujian
155	GT40	2017/07	Lincang, Yunnan	Nanning, Guangxi
156	GT44	2017/07	Lincang, Yunnan	Nanning, Guangxi
157	GT08-1589	2017/07	Lincang, Yunnan	Nanning, Guangxi
158	YT60	2017/07	Lincang, Yunnan	Guangzhou, Guangdong
159	GT02-901	2017/07	Lincang, Yunnan	Nanning, Guangxi
160	LC05-136	2017/07	Lincang, Yunnan	Liuzhou, Guangxi
161	ROC22	2017/07	Lincang, Yunnan	Taiwan Island
162	FN40	2017/07	Lincang, Yunnan	Fuzhou, Fujian
163	YZ05-51	2017/07	Lincang, Yunnan	Kaiyuan, Yunnan
164	GT31	2017/07	Lincang, Yunnan	Nanning, Guangxi
165	YT86-368	2017/07	Lincang, Yunnan	Guangzhou, Guangdong
166	YZ05-49	2017/07	Lincang, Yunnan	Kaiyuan, Yunnan
167	YT93-160	2017/07	Lincang, Yunnan	Guangzhou, Guangdong
168	YT93-160	2017/07	Lincang, Yunnan	Guangzhou, Guangdong
169	YT93-160	2017/07	Lincang, Yunnan	Guangzhou, Guangdong
170	DZ07-78	2017/07	Lincang, Yunnan	Dehong, Yunnan
171	YT60	2017/07	Lincang, Yunnan	Guangzhou, Guangdong
172	YR09-826	2017/07	Lincang, Yunnan	Ruili, Yunnan
173	DZ09-78	2017/07	Lincang, Yunnan	Dehong, Yunnan
174	Dianzhe02-277	2017/07	Lincang, Yunnan	Kaiyuan, Yunnan
175	ROC22	2017/07	Lincang, Yunnan	Taiwan Island
176	YT86-368	2017/07	Lincang, Yunnan	Guangzhou, Guangdong

[a] The number in the bracket presents the order of leaf samples in different plants.
